# Prognostic Factors and Clinical Characteristics of Varicella Zoster Virus Meningitis: Impact of Treatment Delay and Age-Related Differences in a Japanese Tertiary Hospital

**DOI:** 10.3390/neurolint18070130

**Published:** 2026-07-08

**Authors:** Kenta Tasaki, Makoto Hara, Hideto Nakajima

**Affiliations:** Division of Neurology, Department of Medicine, Nihon University School of Medicine, Tokyo 173-8610, Japan; tasaki.kenta@nihon-u.ac.jp (K.T.); nakajima.hideto@nihon-u.ac.jp (H.N.)

**Keywords:** varicella zoster virus meningitis, acyclovir, elderly patients, prognostic factors, treatment delay, oral antiviral agents

## Abstract

**Objectives:** Varicella zoster virus (VZV) meningitis is a complication of herpes zoster that causes high rates of residual symptoms. However, prognostic factors and optimal management strategies remain unclear. This study investigated factors affecting functional outcomes, age-related differences, and the impact of prior oral antiviral therapy in VZV meningitis. **Methods:** This retrospective observational study enrolled patients admitted for aseptic meningitis between 2013 and 2022. The primary outcome was residual symptoms at discharge, defined as a ≥1-point increase in the modified Rankin Scale (mRS) from baseline. Multiple logistic regression identified independent risk factors. **Results:** Among 176 patients with aseptic meningitis, 60 (34.1%) had VZV meningitis. Patients with VZV meningitis had higher rates of residual symptoms (43.3% vs. 12.9%, *p* < 0.001). Independent predictors of residual symptoms included delayed intravenous acyclovir initiation (odds ratio [OR] = 1.303, 95% confidence interval [CI] = 1.060–1.601, *p* = 0.012), corresponding to a 30.3% increase in the odds of residual symptoms for each additional day before treatment initiation, and pre-onset mRS (OR = 2.352, 95% CI = 1.056–5.237, *p* = 0.036). Patients ≥ 50 years old displayed lower rates of headache (75.0% vs. 96.9%, *p* = 0.020), neck stiffness (25.0% vs. 62.5%, *p* = 0.005), and CSF pleocytosis (56/μL vs. 142/μL, *p* = 0.023). Prior oral antiviral therapy was not associated with a rate of residual symptoms (*p* = 0.795). **Conclusions:** Delayed initiation of intravenous acyclovir was independently associated with residual symptoms at discharge, whereas older patients often presented with atypical clinical features, requiring heightened clinical suspicion. Given the lack of observed benefit associated with prior oral antiviral therapy, prompt initiation of intravenous acyclovir should be considered when VZV meningitis is suspected.

## 1. Introduction

Varicella-zoster virus (VZV), also known as Human alphaherpesvirus 3 (HHV-3; species name: Varicellovirus humanalpha3), is a highly neurotropic and exclusively human herpesvirus with a worldwide distribution [1,2,3]. Primary infection causes varicella (chickenpox), whereas viral reactivation results in herpes zoster (shingles) [1,2,3]. VZV is transmitted primarily through respiratory droplets and direct contact with vesicular fluid, remains latent in sensory ganglia following primary infection, and can reactivate later in life [1,2,4]. Although vaccination effectively reduces the incidence of herpes zoster and its complications, breakthrough infections continue to occur, particularly in older adults and immunocompromised individuals [1,3]. Antiviral agents, including acyclovir, valacyclovir, famciclovir, and amenamevir, are widely used to reduce the severity and duration of herpes zoster and its associated complications [3].

VZV meningitis affects thousands of patients annually, yet optimal management remains poorly defined, resulting in persistent disability in nearly half of affected individuals [5,6,7]. Unlike bacterial meningitis, for which prompt antibiotic therapy dramatically improves outcomes, the therapeutic window and treatment imperatives for VZV meningitis have not been established with similar precision. VZV reactivates during the lifetimes of approximately one-third of individuals [1,2,3,4]. Although most cases manifest as uncomplicated herpes zoster, central nervous system (CNS) invasion occurs in 0.5–1% of cases, leading to meningitis or encephalitis with potentially devastating consequences [8,9]. The paradox of VZV meningitis lies in its apparently straightforward treatment using intravenous acyclovir, but the high rate of residual symptoms suggests critical gaps regarding therapeutic timing and patient selection [7,10,11]. In Japan’s rapidly aging society, in which 70% of herpes zoster cases occur in individuals older than 50 years, the burden of VZV meningitis is expected to substantially rise, making the identification of modifiable prognostic factors and evidence-based treatment protocols an urgent clinical priority [12,13]. Despite 10 mg/kg intravenous acyclovir being the recommended standard of care, real-world practice reveals considerable variation, with many patients receiving oral antivirals initially based on cutaneous manifestations, raising fundamental questions regarding whether this approach compromises outcomes or represents acceptable pragmatic management [14].

Despite the substantial clinical burden of VZV meningitis, critical knowledge gaps persist in its management. Although a retrospective study by Yan et al. identified sex and treatment delay as prognostic factors [15], these findings emerged from a different healthcare system, and they lack the necessary granular detail to guide real-world clinical decisions, particularly the quantitative impact of each day of treatment delay [11,14,16]. This therapeutic dilemma is further complicated by age-related heterogeneity in disease presentation and response, but no studies have systematically characterized the manifestation of VZV meningitis across age groups, leaving clinicians without age-specific diagnostic criteria or treatment protocols [9,14,17]. In clinical practice, many patients with herpes zoster receive oral antivirals as initial therapy based on cutaneous manifestations before neurological complications become apparent, but the effects of this common practice on neurological outcomes associated with subsequent meningitis development have not been clarified [17,18]. The absence of data from advanced medical facilities, in which complex cases concentrate and comprehensive diagnostic capabilities exist, further limits our understanding of the full spectrum of VZV meningitis presentations and outcomes.

Therefore, this study examined the clinical characteristics of VZV meningitis in a Japanese tertiary hospital. We investigated factors affecting functional prognosis, quantified the impact of treatment delay, and characterized age-specific presentations. Additionally, we evaluated whether prior oral antiviral therapy influences outcomes when patients progress to meningitis. These findings are expected to establish evidence-based protocols for early recognition and optimal treatment timing in VZV meningitis, ultimately reducing the substantial burden of residual neurological disability.

## 2. Materials and Methods

### 2.1. Study Design and Setting

This single-center, retrospective observational study included patients aged ≥15 years with aseptic meningitis who were admitted to the Department of Neurology at Nihon University Itabashi Hospital, a tertiary referral center, between 1 January 2013, and 31 December 2022. Anonymized pre-existing clinical data were extracted from medical records and systematically analyzed. The study was conducted in accordance with the Declaration of Helsinki and was approved by the Ethics Committee of Nihon University Itabashi Hospital on 22 September 2023 (approval no. RK-230912-12). Owing to the retrospective nature of the study, the requirement for written informed consent from individual patients was waived, and the informed consent process was conducted through an opt-out procedure.

### 2.2. Study Population and Selection Criteria

The inclusion criteria were as follows: presence of meningitis symptoms, such as fever, headache, and neck stiffness; negative cerebrospinal fluid (CSF) bacterial culture; and CSF cell count exceeding 5/μL or identification of a pathogenic virus in CSF. We excluded patients with encephalitis symptoms, such as seizures, severe consciousness disturbance, and psychiatric symptoms, and those with myelitis symptoms, such as motor paralysis and bladder and bowel dysfunction, as their clinical courses differ significantly. Among patients meeting the criteria for aseptic meningitis, a diagnosis of VZV meningitis was indicated by a positive PCR result for VZV DNA in CSF. For comparative analyses, we used patients with aseptic meningitis who did not have VZV detected in CSF, including those who with other identified etiologies and those with an undetermined etiology.

### 2.3. Data Collection

We systematically extracted data from the medical records of all patients admitted with aseptic meningitis during the study period. For each patient, we collected comprehensive clinical information, including characteristics (age, sex, underlying diseases), clinical symptoms (fever, headache, nausea/vomiting, neck stiffness, cranial nerve palsy, skin rash), laboratory findings (CSF cell count and protein concentration), etiology of aseptic meningitis, and treatment details (type of antiviral agent, route of administration, timing of initiation).

### 2.4. Outcome Measures

The primary outcome was the presence of residual symptoms at discharge, defined as a difference of ≥1 in the modified Rankin Scale (mRS) scores between before onset and discharge. mRS is a standardized scale evaluating neurological disability on a scale from 0 (no symptoms) to 6 (death). Residual symptoms were not limited to specific manifestations and were assessed by the attending physician using the mRS, based on both subjective patient-reported symptoms and objective neurological findings. This definition enabled an objective assessment of functional decline attributable to VZV meningitis relative to baseline status. Secondary outcomes included clinical differences between age groups (≥50 years vs. <50 years; the cutoff of 50 years was selected because it corresponds to the recommended age threshold for herpes zoster vaccination in Japan), the impact of prior oral antiviral therapy (acyclovir, valacyclovir, famciclovir, or amenamevir) compared with initial intravenous acyclovir treatment, length of hospital stay, interval from onset to intravenous acyclovir initiation, interval from onset to resolution of meningitis symptoms, and CSF findings. Neurological function was assessed using mRS at three time points: pre-onset (evaluated retrospectively), peak severity during hospitalization, and discharge. We also recorded the duration from meningitis symptom onset to resolution to evaluate recovery.

### 2.5. Statistical Analysis

Continuous variables were presented as the median and range based on their non-normal distribution, whereas categorical variables were presented as numbers and percentages. The Mann–Whitney *U* test was used to compare continuous variables between groups, and Fisher’s exact test was used for between-group comparisons of categorical variables. Multiple logistic regression analysis identified factors affecting residual symptoms at discharge, with age, sex, underlying diseases, time to intravenous acyclovir initiation, and pre-onset mRS serving as independent variables. These variables were selected a priori based on their clinical relevance and potential confounding effects. Clinical manifestations and CSF parameters were not included because of the limited sample size and the exploratory nature of the analysis, whereas variables reflecting disease severity during hospitalization or at discharge, such as peak and discharge mRS scores, were excluded from the model. Model fit was evaluated using the chi-squared and Hosmer–Lemeshow tests, and the discrimination rate was calculated. Statistical significance was denoted by *p* < 0.05. Statistical analyses were performed using IBM SPSS Statistics (version 28.0.0; IBM Corp., Armonk, NY, USA) and GraphPad Prism (version 10.6.1; GraphPad Software, Boston, MA, USA).

## 3. Results

### 3.1. Patient Characteristics and Etiology of Aseptic Meningitis

**Figure 1:**
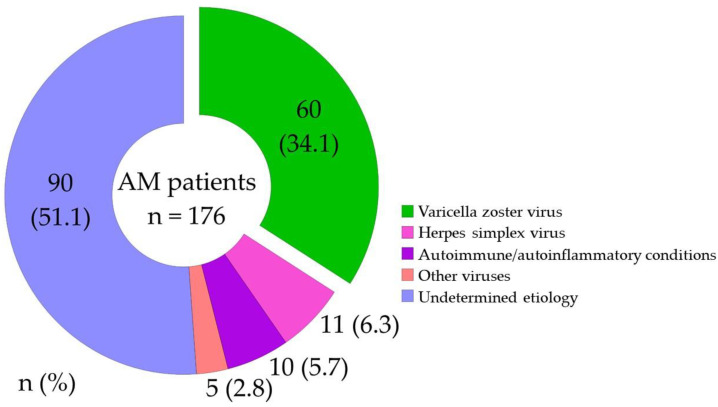
Etiological distribution of aseptic meningitis (AM) in 176 patients. Varicella zoster virus was the most common identifiable pathogen (34.1%), followed by herpes simplex virus (6.3%), autoimmune/autoinflammatory conditions (5.7%), and other viruses (2.8%). The etiology remained undetermined in 51.1% of patients despite comprehensive diagnostic workup including viral PCR testing in cerebrospinal fluid.

During the 10-year study period, 176 patients with aseptic meningitis were admitted to our department. VZV was identified as the causative pathogen in 60 patients (34.1%), representing the most common identifiable cause of aseptic meningitis in our cohort (Figure 1). The etiology remained undetermined in 90 patients (51.1%), whereas other identified causes included herpes simplex virus in 11 patients (6.3%), autoimmune or autoinflammatory conditions in 10 patients (5.7%), and other viruses in 5 patients (2.8%).

### 3.2. Comparison Between VZV Meningitis and Other Forms of Aseptic Meningitis

Patients with VZV meningitis displayed distinct clinical characteristics from those with other causes of aseptic meningitis (Table 1). The VZV meningitis group had a significantly higher median age (46 years; range, 18–97) than the other aseptic meningitis group (34 years; range, 15–85; *p* < 0.001), in addition to a higher proportion of patients with underlying diseases (38.3% vs. 15.5%, *p* = 0.001). Regarding clinical manifestations, fever was less common in the VZV meningitis group (78.3% vs. 93.1%, *p* = 0.006), whereas cranial nerve palsy occurred more frequently (16.6% vs. 4.3%, *p* = 0.009). Notably, skin rash was present in 86.7% of patients in the VZV meningitis group, versus only 5.2% of those in the other aseptic meningitis group (*p* < 0.001), representing a distinctive clinical marker. Although CSF cell counts and protein levels did not significantly differ between the groups, the clinical course differed substantially. The median interval from onset to resolution of meningitis symptoms was shorter in the VZV meningitis group (10 days vs. 12 days, *p* = 0.037), yet these patients experienced longer median hospital stays (20 days vs. 15 days, *p* < 0.001) and a significantly higher rate of residual symptoms at discharge (43.3% vs. 12.9%, *p* < 0.001). The pre-onset and discharge mRS scores were also significantly worse in the VZV meningitis group (*p* = 0.003 and *p* < 0.001, respectively), indicating greater functional impairment. Recurrence was not observed in the VZV meningitis group, whereas 7.8% of patients in the other aseptic meningitis group experienced recurrence (*p* = 0.029).

### 3.3. Age-Related Differences in VZV Meningitis

Analyses of age-related differences revealed distinct clinical patterns between older and younger patients with VZV meningitis (Table 2). Patients aged ≥50 years (n = 28) included a significantly higher proportion of female patients (67.9% vs. 34.4%, *p* = 0.019) and a higher rate of underlying diseases (60.7% vs. 18.8%, *p* = 0.001) than those aged < 50 years (n = 32). Older patients were less likely to present with typical meningitis symptoms, including headache (75.0% vs. 96.9%, *p* = 0.020) and neck stiffness (25.0% vs. 62.5%, *p* = 0.005). CSF analysis revealed significantly lower median cell counts in the older group (56/μL vs. 142/μL, *p* = 0.023), while CSF protein levels remained comparable between age groups. Although the median interval from onset to intravenous acyclovir initiation was comparable between the groups (5 days vs. 6 days, *p* = 0.815), older patients had significantly worse pre-onset (*p* = 0.001) and peak mRS scores during hospitalization (*p* = 0.003). The median length of hospital stay was significantly longer in older patients (23 days vs. 18 days, *p* < 0.001), but the rate of residual symptoms at discharge did not differ significantly between these groups (46.4% vs. 40.6%, *p* = 0.795).

### 3.4. Factors Associated with Residual Symptoms at Discharge

Among 60 patients with VZV meningitis, 26 (43.3%) had residual symptoms at discharge (Table 3). Univariate analysis revealed that patients with residual symptoms had a significantly longer median interval from onset to intravenous acyclovir initiation (7 days vs. 5 days, *p* = 0.045). Although patients with residual symptoms tended to have higher rates of cranial nerve palsy (26.9% vs. 8.8%, *p* = 0.085) and worse peak mRS scores during hospitalization (*p* = 0.073), these differences did not reach statistical significance. The median discharge mRS was significantly higher in patients with residual symptoms (1 vs. 0, *p* < 0.001), and their median hospital stay was significantly longer (22 days vs. 19 days, *p* = 0.009). Age, sex, underlying diseases, or CSF findings did not significantly differ between patients with and without residual symptoms (Table 3). Multiple logistic regression analysis identified the interval from onset to intravenous acyclovir initiation (odds ratio [OR] = 1.303, 95% confidence interval [CI] = 1.060–1.601, *p* = 0.012), indicating a 30.3% increase in the odds of residual symptoms for each additional day before treatment initiation, and pre-onset mRS (OR = 2.352, 95% CI = 1.056–5.237, *p* = 0.036) as independent risk factors for residual symptoms at discharge (Table 4). The model displayed good fit with a chi-squared test *p*-value of 0.002, Hosmer–Lemeshow test *p*-value of 0.201, and discrimination accuracy of 70.0%.

### 3.5. Impact of Prior Oral Antiviral Therapy

Of the 60 patients with VZV meningitis, 26 (43.3%) received oral antiviral therapy before starting intravenous acyclovir treatment, whereas 34 (56.7%) were initially treated with intravenous acyclovir (Table 5). The oral antivirals used included acyclovir in 2 patients (7.7%), valacyclovir in 9 patients (34.6%), famciclovir in 11 patients (42.3%), and amenamevir in 4 patients (15.4%). Patients who received prior oral antiviral therapy were significantly older than those who received initial intravenous treatment (63 years vs. 41 years, *p* = 0.007), and the rate of skin rash was higher in patients who received prior oral antiviral therapy (100.0% vs. 76.5%, *p* = 0.008). Despite the theoretical concerns about the limited bioavailability and CSF penetration of oral antivirals, there were no significant between-group differences in the median interval from onset to intravenous acyclovir initiation (5 days vs. 6 days, *p* = 0.808), median interval from onset to resolution of meningitis symptoms (7 days vs. 10 days, *p* = 0.722), or median length of hospital stay (21 days vs. 20 days, *p* = 0.501). Most importantly, the rate of residual symptoms at discharge was similar between patients who did and did not receive prior oral therapy (46.2% vs. 41.2%, *p* = 0.795), and functional outcomes as measured by discharge mRS did not differ between these groups (1 vs. 0, *p* = 0.569).

## 4. Discussion

In this 10-year retrospective study, we analyzed the clinical characteristics of patients with VZV meningitis encountered at a designated hospital for advanced treatment, focusing on age-related differences, prognostic factors, and the impact of prior oral antiviral therapy. Our study revealed three key findings. First, early initiation of intravenous acyclovir and baseline functional status were independent predictors of functional outcomes at discharge. Second, older patients with VZV meningitis exhibited less pronounced symptoms, potentially complicating diagnosis and prolonging hospitalization. Third, prior oral antiviral therapy did not adversely affect clinical outcomes when followed by appropriate intravenous treatment. These findings provide important insights for optimizing the management of VZV meningitis in clinical practice.

Our finding that delayed intravenous acyclovir initiation independently predicts residual symptoms at discharge provides crucial evidence supporting the importance of timely treatment in VZV meningitis. In our cohort, each day of delay increased the odds of residual symptoms by 30.3%. This aligns with and extends the findings of Yan et al., who identified treatment delay as an independent prognostic factor in their single-center study, although they did not quantify the specific impact per day of delay [15]. Our results also identified pre-onset functional status as an independent predictor, a factor not previously reported in the VZV meningitis literature. Although previous studies recommended prompt antiviral therapy based on expert opinion [18,19], our study provides quantitative evidence supporting this recommendation. The association between treatment delay and poor outcomes likely reflects ongoing viral replication and neuronal damage during the untreated period, as VZV can cause direct cytopathic effects and inflammatory responses in the CNS [20,21,22]. These findings illustrate that VZV meningitis should be considered a medical urgency requiring immediate intravenous acyclovir upon clinical suspicion opposed to awaiting confirmatory laboratory results, particularly in patients with pre-existing functional impairment, who face a higher risk of residual disability [20,21,22].

Our study demonstrated that older patients with VZV meningitis have distinct clinical features from those of younger patients. These findings extend previous observations that the incidence of herpes zoster increases with age, suggesting that the clinical presentation of VZV meningitis may change with aging, with older patients showing less pronounced CSF inflammatory findings. Whereas previous studies noted that older patients can have atypical presentations of infectious diseases in general [23,24], our study is the first to systematically document these specific differences in VZV meningitis. The attenuated inflammatory response and subtle clinical manifestations in older patients likely reflect age-related immunosenescence (progressive deterioration of immune function with aging), which affects both innate and adaptive immune responses [25,26]. This atypical presentation has critical clinical implications, as it can lead to diagnostic delays and misdiagnosis, particularly when physicians rely on classic meningitis symptoms for clinical decision-making [9,14,23,24]. Our finding of significantly longer hospital stays in older patients despite similar treatment protocols suggests that these diagnostic challenges translate into real clinical consequences. These results emphasize the need for heightened clinical suspicion for VZV meningitis in older patients presenting with unexplained neurological symptoms, even in the absence of typical meningitis signs.

Our finding that a diagnosis of herpes zoster prompted early oral antiviral treatment, but that prior oral antiviral therapy did not improve clinical outcomes in VZV meningitis, provides important insights into the pathophysiology and treatment requirements of this condition. This lack of benefit occurred even though all patients in the prior oral antiviral therapy group had visible skin rash at presentation, indicating they were diagnosed and treated at the herpes zoster stage. These results suggest that VZV has likely invaded the CNS by the time herpes zoster manifests in patients who later develop meningitis [20,27,28], and oral antivirals with limited CSF penetration, such as valacyclovir [29,30] and amenamevir [31], cannot prevent or slow the progression to meningitis. This finding aligns with the known pathophysiology of VZV reactivation, in which viral spread from sensory ganglia can occur through both peripheral nerves to the skin and centrally to the meninges simultaneously [20,28,32,33]. Previous studies focused on optimal treatment regimens for established VZV meningitis, but the ability of early oral intervention during the herpes zoster phase to prevent CNS complications has not been addressed [14,20,34,35]. Our data suggest that oral antiviral therapy did not improve the outcomes of VZV meningitis. Most critically, our finding that delayed intravenous acyclovir initiation was independently associated with residual symptoms at discharge emphasizes the importance of prompt recognition and treatment of suspected VZV meningitis. However, because detailed information regarding the dosage and duration of prior oral antiviral therapy was not consistently available, the effectiveness of oral antiviral agents could not be evaluated.

Among oral antivirals for herpes zoster, amenamevir warrants particular attention because of its unique pharmacological profile. Unlike valacyclovir, for which CSF levels only reach approximately 50% of plasma levels [29,30], amenamevir demonstrates virtually no CSF penetration [31], raising theoretical concerns about the CNS complications of VZV. Recent case reports by Taniguchi et al. and Itoh et al. described aseptic meningitis in patients receiving amenamevir for trigeminal herpes zoster [36,37]. Tsumura et al. found that 57% of patients who developed VZV meningitis/encephalitis had received amenamevir, compared with 28% in the general population [17]. Although a large-scale analysis at the Japan Medical Data Center claims database found no significant difference in CNS infection outcomes between amenamevir and other antivirals [17], this could reflect limitations in claims data rather than true equivalence. The discrepancy between theoretical pharmacokinetic concerns and population-level outcomes aligns with our findings that prior oral therapy, regardless of agent, failed to prevent meningitis progression in more than 40% of patients. Critically, our data and emerging evidence reinforce that when VZV meningitis is suspected following treatment with amenamevir or any oral antiviral, immediate intravenous acyclovir remains critical, as the CNS penetration of oral agents is insufficient to treat established infection.

This single-center retrospective study conducted at a tertiary referral hospital had several important limitations. First, our findings might not be generalizable to other healthcare settings, as patients referred to our designated hospital for advanced treatment could represent more complex or severe cases. Second, diagnosis relied on PCR-based detection of VZV DNA in CSF, potentially missing cases with low viral loads or those tested after viral clearance, leading to underestimation of the prevalence of VZV meningitis. Third, the exclusion of patients with encephalitis or myelitis symptoms might have eliminated severe cases in which meningitis progressed to extensive CNS involvement. Fourth, retrospective assessment of pre-onset functional status based on medical records might have introduced recall bias, particularly for subtle pre-existing disabilities. Fifth, we did not systematically evaluate long-term outcomes beyond hospital discharge, as post-discharge follow-up data were available for only a subset of patients. Therefore, persistent neurological sequelae and long-term functional outcomes could not be adequately assessed. Sixth, neuroimaging findings, particularly magnetic resonance imaging (MRI) abnormalities that have been associated with prognosis in previous studies, were not included in the analysis because MRI was not performed systematically in all patients [20,28,32]. Seventh, although VZV DNA PCR testing was performed using a quantitative assay, viral load data were not consistently available as standardized continuous measurements in this retrospective study. Therefore, we could not evaluate the association between CSF VZV DNA levels and clinical outcomes, despite previous studies suggesting a potential prognostic role of viral burden [22]. Eighth, inconsistent documentation of prior oral antiviral therapy duration and dosing prevented detailed analyses of treatment exposure and dose–response relationships. Finally, our sample size of 60 patients with VZV meningitis, although substantial for a single center, might have been insufficient to detect clinically meaningful differences in some subgroup analyses. Despite these limitations, our study provides valuable real-world insights into VZV meningitis clinical characteristics and prognostic factors, emphasizing the critical importance of early diagnosis and prompt intravenous antiviral therapy in preventing functional decline.

## 5. Conclusions

This study provides important evidence for VZV meningitis management. Delayed initiation of intravenous acyclovir was independently associated with risk of residual symptoms at discharge, with an approximately 30% higher odds for each additional day before treatment initiation. Older patients presented atypically with reduced meningitis signs and attenuated CSF responses, requiring heightened clinical suspicion. Prior oral antiviral therapy provided no apparent clinical benefit. Based on these findings, low diagnostic thresholds should be maintained in older patients and treatment should be initiated promptly without awaiting confirmatory test results. Future prospective studies are needed to validate these findings and establish causal relationship.

## Figures and Tables

**Table 1:** Comparison of clinical characteristics between the VZV meningitis and other aseptic meningitis groups.

	VZV Meningitis (n = 60)	Other Aseptic Meningitis (n = 116)	*p*
**Demographics and clinical characteristics**			
Age, years, median [range]	46 [18–97]	34 [15–85]	<0.001
Sex, male:female, n (%)	30:30 (50.0:50.0)	48:68 (41.4:58.6)	0.337
Underlying disease, n (%)	23 (38.3)	18 (15.5)	0.001
**Clinical symptoms**			
Meningitis symptoms			
Fever, n (%)	47 (78.3)	108 (93.1)	0.006
Headache, n (%)	52 (86.7)	108 (93.1)	0.175
Nausea/vomiting, n (%)	30 (50.0)	62 (53.4)	0.751
Neck stiffness, n (%)	27 (45.0)	52 (42.8)	>0.999
Cranial nerve palsy, n (%)	10 (16.6)	5 (4.3)	0.009
Skin rash, n (%)	52 (86.7)	6 (5.2)	<0.001
**CSF findings**			
CSF cell count, /μL, median [range]	104 [1–952]	72 [6–700]	0.606
CSF protein, mg/dL, median [range]	77 [32–1812]	70 [20–300]	0.120
**Treatment**			
Interval from onset to intravenous acyclovir initiation, days, median [range]	5 [1–22]	5 [0–22]	0.806
Prior oral antiviral therapy, n (%)	26 (43.3)	NA	
**Functional status (mRS)**			
Pre-onset mRS, median [range]	0 [0–4]	0 [0–2]	0.003
Peak mRS, median [range]	2 [1–5]	2 [1–5]	0.051
Discharge mRS, median [range]	0 [0–5]	0 [0–3]	<0.001
**Outcomes**			
Interval from onset to resolution of meningitis symptoms, days, median [range]	10 [1–30]	12 [0–36]	0.037
Length of hospital stay, days, median [range]	20 [10–51]	15 [4–62]	<0.001
Residual symptoms at discharge, n (%)	26 (43.3)	15 (12.9)	<0.001
Recurrence, n (%)	0 (0.0)	9 (7.8)	0.029

Some patients in the other aseptic meningitis group also received intravenous acyclovir empirically. VZV, varicella zoster virus; CSF, cerebrospinal fluid; mRS, modified Rankin Scale.

**Table 2:** Clinical characteristics of patients with varicella zoster virus meningitis by age group.

	≥50 Years (n = 28)	<50 Years (n = 32)	*p*
**Demographics and clinical characteristics**			
Sex, male:female, n (%)	9:19 (32.1:67.9)	21:11 (65.6:34.4)	0.019
Underlying disease, n (%)	17 (60.7)	6 (18.8)	0.001
**Clinical symptoms**			
Meningitis symptoms			
Fever, n (%)	20 (71.4)	27 (84.4)	0.347
Headache, n (%)	21 (75.0)	31 (96.9)	0.020
Nausea/vomiting, n (%)	12 (42.9)	18 (56.3)	0.438
Neck stiffness, n (%)	7 (25.0)	20 (62.5)	0.005
Cranial nerve palsy, n (%)	5 (17.9)	5 (15.6)	>0.999
Skin rash, n (%)	25 (89.3)	27 (84.4)	0.712
**CSF findings**			
CSF cell count, /μL, median [range]	56 [1952]	142 [7–463]	0.023
CSF protein, mg/dL, median [range]	76 [35–1812]	78 [32–313]	0.697
**Treatment**			
Interval from onset to intravenous acyclovir initiation, days, median [range]	5 [1–22]	6 [2–13]	0.815
Prior oral antiviral therapy, n (%)	16 (57.1)	10 (31.3)	0.067
**Functional status (mRS)**			
Pre-onset mRS, median [range]	0 [0–4]	0 [0–0]	0.001
Peak mRS, median [range]	2 [1–5]	2 [1–4]	0.003
Discharge mRS, median [range]	1 [0–5]	0 [0–1]	0.060
**Outcomes**			
Interval from onset to resolution of meningitis symptoms, days, median [range]	6 [1–20]	10 [2–30]	0.127
Length of hospital stay, days, median [range]	23 [15–51]	18 [10–22]	<0.001
Residual symptoms at discharge, n (%)	13 (46.4)	13 (40.6)	0.795

CSF, cerebrospinal fluid; mRS, modified Rankin Scale.

**Table 3:** Comparison of patients with varicella zoster virus meningitis based on the presence of residual symptoms at discharge.

	Residual Symptoms (n = 26)	No Residual Symptoms (n = 34)	*p*
**Demographics and clinical characteristics**			
Age, years, median [range]	50 [23–97]	44 [18–84]	0.392
Sex, male:female, n (%)	12:14 (46.2:53.8)	18:16 (52.9:47.1)	0.795
Underlying disease, n (%)	11 (42.3)	12 (35.3)	0.603
**Clinical symptoms**			
Meningitis symptoms			
Fever, n (%)	19 (73.1)	28 (82.4)	0.529
Headache, n (%)	20 (76.9)	32 (94.1)	0.067
Nausea/vomiting, n (%)	11 (42.3)	19 (55.9)	0.435
Neck stiffness, n (%)	11 (42.3)	16 (47.1)	0.796
Cranial nerve palsy, n (%)	7 (26.9)	3 (8.8)	0.085
Skin rash, n (%)	23 (88.5)	29 (85.3)	>0.999
**CSF findings**			
CSF cell count, /μL, median [range]	96 [1–952]	104 [2–463]	0.352
CSF protein, mg/dL, median [range]	82 [32–1812]	77 [36–313]	0.708
**Treatment**			
Interval from onset to intravenous acyclovir initiation, days, median [range]	7 [2–22]	5 [1–10]	0.045
Prior oral antiviral therapy, n (%)	12 (46.2)	14 (41.2)	0.795
**Functional status (mRS)**			
Pre-onset mRS, median [range]	0 [0–4]	0 [0–2]	0.127
Peak mRS, median [range]	2 [1–5]	2 [1–4]	0.074
Discharge mRS, median [range]	1 [1–5]	0 [0–1]	<0.001
**Outcomes**			
Interval from onset to resolution of meningitis symptoms, days, median [range]	11 [1–30]	9 [2–19]	0.378
Length of hospital stay, days, median [range]	22 [15–51]	19 [10–29]	0.009

CSF, cerebrospinal fluid; mRS, modified Rankin Scale.

**Table 4:** Multiple logistic regression analysis for residual symptoms at discharge in patients with varicella zoster virus meningitis.

Variables	β	SE	Wald	OR [95% CI]	*p*
Days to intravenous acyclovir initiation	0.265	0.105	6.341	1.303 [1.060–1.601]	0.012
Pre-onset mRS	0.855	0.408	4.382	2.352 [1.056–5.237]	0.036

Dependent variable: Presence of residual symptoms at discharge. Independent variables: age, sex, presence of underlying diseases, days from symptom onset to intravenous acyclovir initiation, and pre-onset modified Rankin Scale. Model chi-squared test: *p* = 0.002; Hosmer–Lemeshow test: *p* = 0.201. SE, standard error; OR, odds ratio; CI, confidence interval.

**Table 5:** Comparison of patients with varicella zoster virus meningitis by initial antiviral treatment approach.

	Prior Oral Antiviral Therapy (n = 26)	Direct Intravenous Acyclovir (n = 34)	*p*
**Demographics and clinical characteristics**			
Age, years, median [range]	63 [22–97]	41 [18–83]	0.007
Sex, male:female, n (%)	9:17 (34.6:65.4)	21:13 (61.8:38.2)	0.067
Underlying disease, n (%)	8 (30.8)	15 (44.1)	0.422
**Clinical symptoms**			
Meningitis symptoms			
Fever, n (%)	20 (76.9)	27 (79.4)	>0.999
Headache, n (%)	20 (76.9)	32 (94.1)	0.069
Nausea/vomiting, n (%)	15 (57.7)	15 (44.1)	0.435
Neck stiffness, n (%)	9 (34.6)	18 (52.9)	0.196
Cranial nerve palsy, n (%)	2 (7.7)	8 (23.5)	0.163
Skin rash, n (%)	26 (100.0)	26 (76.5)	0.008
**CSF findings**			
CSF cell count, /μL, median [range]	87 [1–952]	117 [2–463]	0.156
CSF protein, mg/dL, median [range]	78 [34–1812]	77 [32–313]	0.782
**Treatment**			
Oral antivirals			
Acyclovir, n (%)	2 (7.7)	-	
Valacyclovir, n (%)	9 (34.6)	-	
Famciclovir, n (%)	11 (42.3)	-	
Amenamevir, n (%)	4 (15.4)	-	
Interval from onset to intravenous acyclovir initiation, days, median [range]	5 [1–14]	6 [1–22]	0.808
**Functional status (mRS)**			
Pre-onset mRS, median [range]	0 [0–4]	0 [0–2]	0.133
Peak mRS, median [range]	2 [1–4]	2 [1–4]	0.059
Discharge mRS, median [range]	1 [0–5]	0 [0–4]	0.569
**Outcomes**			
Interval from onset to resolution of meningitis symptoms, days, median [range]	7 [1–20]	10 [1–30]	0.722
Length of hospital stay, days, median [range]	21 [15–51]	20 [10–44]	0.501
Residual symptoms at discharge, n (%)	12 (46.2)	14 (41.2)	0.795

CSF, cerebrospinal fluid; mRS, modified Rankin Scale.

## Data Availability

The datasets generated and analyzed during the current study are available from the corresponding author upon request.

## Abbreviations

The following abbreviations are used in this manuscript:

VZV	Varicella Zoster Virus
mRS	modified Rankin Scale
OR	Odds Ratio
CI	Confidence Interval
CNS	Central Nervous System
CSF	Cerebrospinal Fluid
SE	Standard Error
